# Rice *OsAAA-ATPase1* is Induced during Blast Infection in a Salicylic Acid-Dependent Manner, and Promotes Blast Fungus Resistance

**DOI:** 10.3390/ijms21041443

**Published:** 2020-02-20

**Authors:** Xinqiong Liu, Haruhiko Inoue, Xianying Tang, Yanping Tan, Xin Xu, Chuntai Wang, Chang-Jie Jiang

**Affiliations:** 1 College of Life Science, South-Central University for Nationalities, Wuhan 430074, China; 2 Institute of Agrobiological Sciences (NIAS), National Agriculture and Food Research Organization (NARO), Tsukuba 305-8602, Japan

**Keywords:** AAA-ATPase, salicylic acid, fatty acid, rice, *Magnaporthe oryzae*, disease resistance

## Abstract

Fatty acids (FAs) have been implicated in signaling roles in plant defense responses. We previously reported that mutation or RNAi-knockdown (*OsSSI2*-kd) of the rice *OsSSI2* gene, encoding a stearoyl acyl carrier protein FA desaturase (SACPD), remarkably enhanced resistance to blast fungus *Magnaporthe oryzae* and the leaf-blight bacterium *Xanthomonas oryzae* pv. *oryzae* (*Xoo*). Transcriptomic analysis identified six AAA-ATPase family genes (hereafter *OsAAA-ATPase1–6*) upregulated in the *OsSSI2*-kd plants, in addition to other well-known defense-related genes. Here, we report the functional analysis of *OsAAA-ATPase1* in rice’s defense response to *M. oryzae*. Recombinant OsAAA-ATPase1 synthesized in *Escherichia coli* showed ATPase activity. *OsAAA-ATPase1* transcription was induced by exogenous treatment with a functional analogue of salicylic acid (SA), benzothiadiazole (BTH), but not by other plant hormones tested. The transcription of *OsAAA-ATPase1* was also highly induced in response to *M. oryzae* infection in an SA-dependent manner, as gene induction was significantly attenuated in a transgenic rice line expressing a bacterial gene (*nahG*) encoding salicylate hydroxylase. Overexpression of *OsAAA-ATPase1* significantly enhanced pathogenesis-related gene expression and the resistance to *M. oryzae*; conversely, RNAi-mediated suppression of this gene compromised this resistance. These results suggest that *OsAAA-APTase1* plays an important role in SA-mediated defense responses against blast fungus *M. oryzae*.

## 1. Introduction

Salicylic acid (SA) plays an important signaling role in plant defense activation against pathogens. In response to pathogen attack, SA activates a battery of defense-related genes, including pathogenesis-related (PR) genes, throughout the plant, resulting in both local and systemic resistance to the pathogen [1]. In *Arabidopsis*, NPR1 (non-pathogenesis related 1) has been demonstrated to play a master role in SA-mediated defense activation [2,3]. A loss of NPR1 function (npr1) results in loss of PR gene induction, and hypersensitivity to diseases [4]. In rice, meanwhile, it has been shown that SA signaling is mediated by two downstream factors, OsNPR1 and WRKY45, acting in parallel [5,6].

In *Arabidopsis*, genetic screening for mutations that can suppress *npr1* phenotypes (based on their ability to restore SA-induced PR expression to npr1-5 plants) resulted in isolation of several *npr1* suppressor mutants (*ssi*: suppressor of SA insensitivity), which exhibit constitutive defense activation [7]. Map-based cloning of one of the *ssi* mutants (*ssi2*) revealed that the corresponding gene (*SSI2*) encodes a stearoyl-ACP desaturase, which desaturates stearoyl (18:0)-ACP into oleoyl-ACP, and finally, into oleic acid (18:1) [7]. Disruption of this gene in *ssi2* results in a ten-fold increase in 18:0 fatty acid (FA) content, indicating involvement of FAs in plant defense reactions [7]. The *ssi2* mutant plants accumulate high levels of SA and display constitutive PR gene expression and enhanced resistance to *Peronospora parasitica*, *Pseudomonas syringae* [8], and *Cucumber mosaic virus* [7,9,10].

The orthologs of *SSI2* have also been identified in soybean (*GmSACPD-A/-B*) [11], rice (*OsSSI2*) [12], and wheat (*TaSSI2*) [13,14]. Similar to *Arabidopsis ssi2*, suppression of these ortholog genes enhanced resistance to multiple pathogens: *Pseudomonas syringae* pv. *glycinea* and *Phytophthora sojae* in soybean [11]; blast fungus *Magnaporthe oryzae* and leaf-blight bacteria *Xanthomonas oryzae* pv. *oryzae* (*Xoo*) in rice [12]; and powdery mildew bacteria *Blumeria graminis* f. sp. *tritici* and Fusarium head blight fungus *Fusarium graminearum* in wheat [13,14]. These results demonstrate a common function of *SSI2* and its orthologs in defense activation in diverse plant species.

The molecular mechanisms whereby the *SSI2* family genes participate in defense reactions in plants remain to be fully elucidated. In rice, a DNA microarray analysis revealed several hundred genes differentially expressed between the wild-type and *OsSSI2*-suppressed transgenic (*OsSSI2*-kd) plants [12]. Among them was a group of six genes for AAA-ATPase (AAA: ATPases associated with diverse cellular activities) highly upregulated in *OsSSI2*-kd plants, in addition to the well-known defense-related genes, such as *WRKY45*, *PR1b*, and *PBZ1*, and a *thaumatin-like* gene [12]. These results suggest that the AAA-ATPase family genes may play important roles in defense activation in rice plants.

The AAA-ATPase family occurs in all life forms, including eukaryotes, prokaryotes, and archaebacteria, and is implicated in a variety of cellular activities, including proteolysis, protein folding, membrane trafficking, cytoskeletal regulation, organelle biogenesis, DNA replication, and immune responses [15,16,17]. Structurally, these proteins contain one or several conserved motifs, including the Walker A and Walker B motifs, which are, respectively, required for ATP binding and hydrolysis; they also contain a highly conserved amino acid sequence, referred to as the second region of homology (SRH) [16]. In plants, it has been reported that AAA-ATPase genes from *Nicotiana tabacum* (*NtAAA1*) [18,19] and *Arabidopsis* (*AtOM66*) [20] are, respectively, negatively or positively involved in the SA-signaling pathway and in the hypersensitive response (HR) upon pathogen infections. Moreover, in rice, map-based cloning of the *lesion mimic resembling* (*lmr*) mutant/*lesion resembling disease* (*lrd6-6*) mutant revealed that the corresponding gene (*LMR/LRD6-6, Os06g0130000*) encodes an AAA-ATPase, and is negatively involved in HR and disease resistance [21,22].

In this study, we conducted a functional analysis of *OsAAA-ATPase1*, one of the six AAA-ATPase genes upregulated in *OsSSI2*-kd rice plants [12]. We show that *OsAAA-ATPase1* is transcriptionally regulated by SA, and positively involved in resistance to blast fugus *M. oryzae*.

## 2. Results

**Figure 1:**
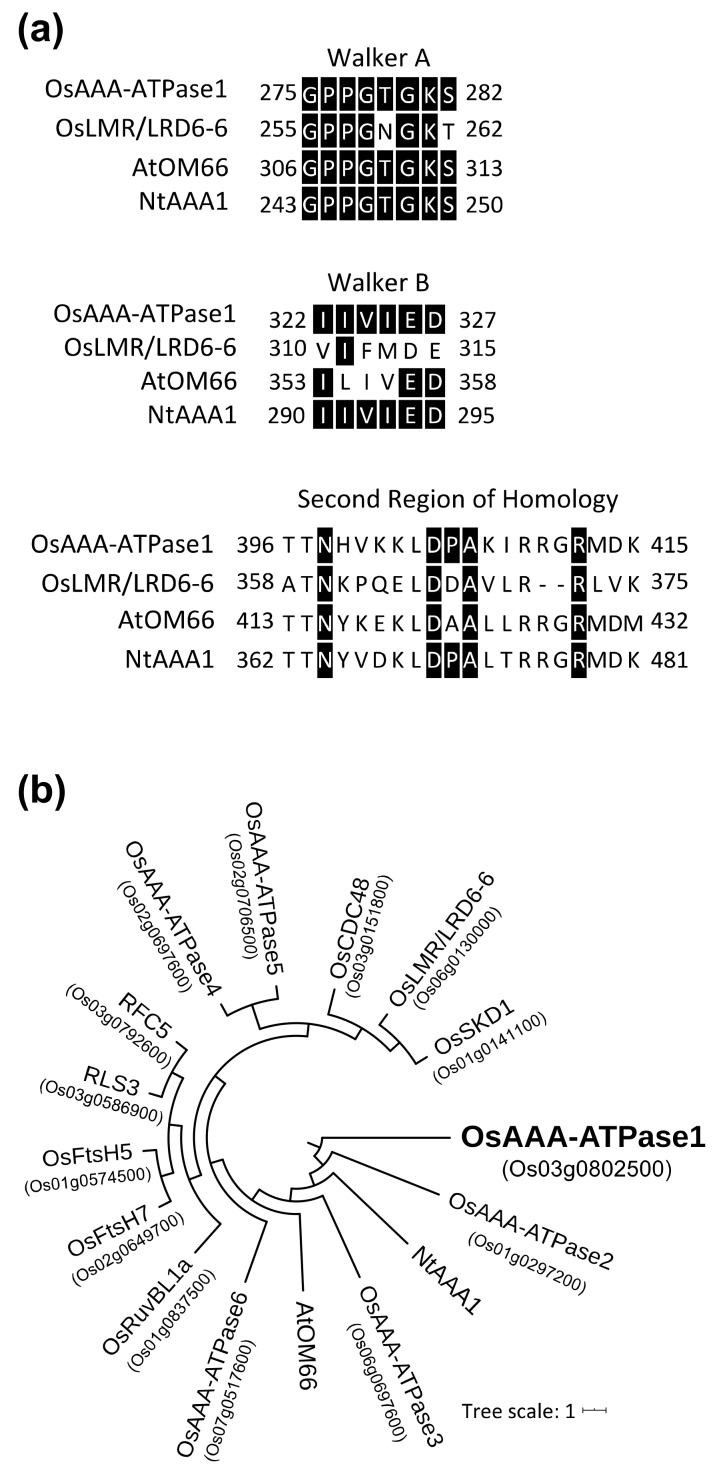
Amino acid sequence alignment of AAA-type ATPase proteins, and phylogenetic analysis. (**a**) Alignment of typical consensus motifs of the AAA-ATPase protein family, including the Walker A, Walker B, and SRH motifs. (**b**) Phylogeny of the AAA-ATPase proteins from rice (LMR/LRD6-6, OsCDC48, RuvBL1a, RLS3, OsSKD1, OsFtsH5, and RFC5), tobacco (NtAAA1), and *Arabidopsis* (AtOM66).

**Figure 2:**
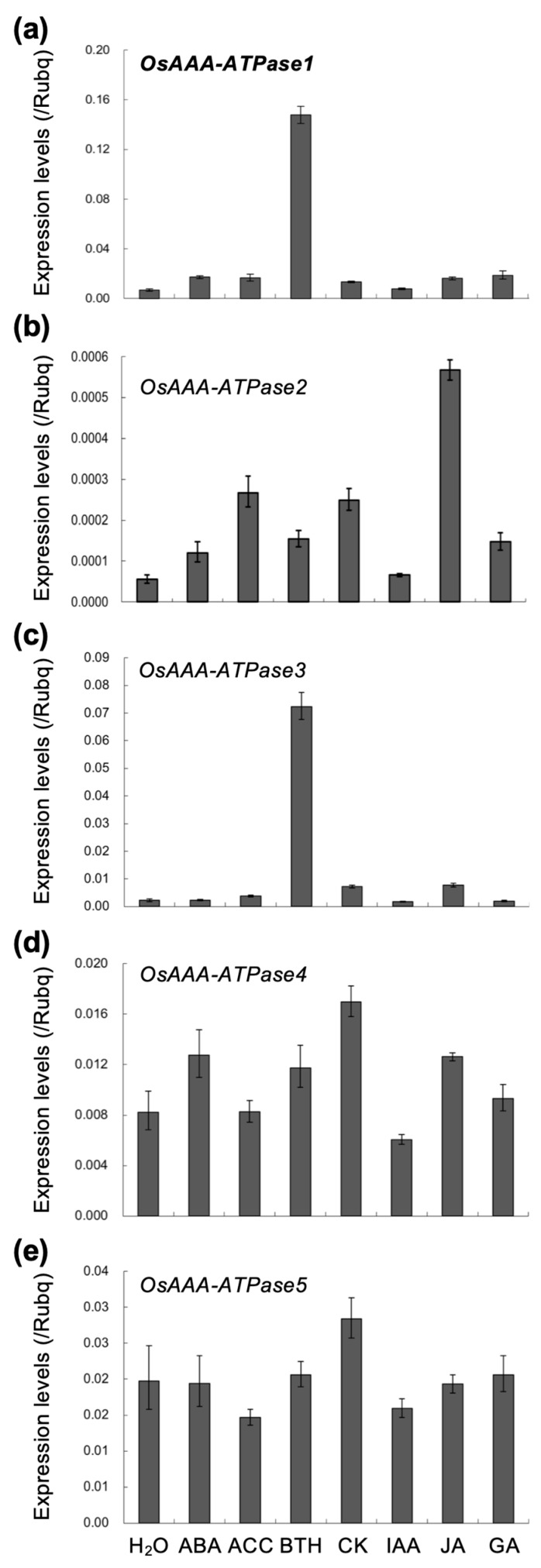
Expression analysis of *OsAAA-ATPase1–5* (**a**–**e**) in response to the plant hormones abscisic acid (ABA), ethylene (ACC, an ethylene precursor), benzothiadiazole (BTH, a functional analogue of SA), kinetin (CK, a synthetic cytokinin), auxin (IAA), jasmonic acid (JA), and gibberellic acid (GA), in Nipponbare rice seedlings. Data are represented as means ± SDs.

**Figure 3:**
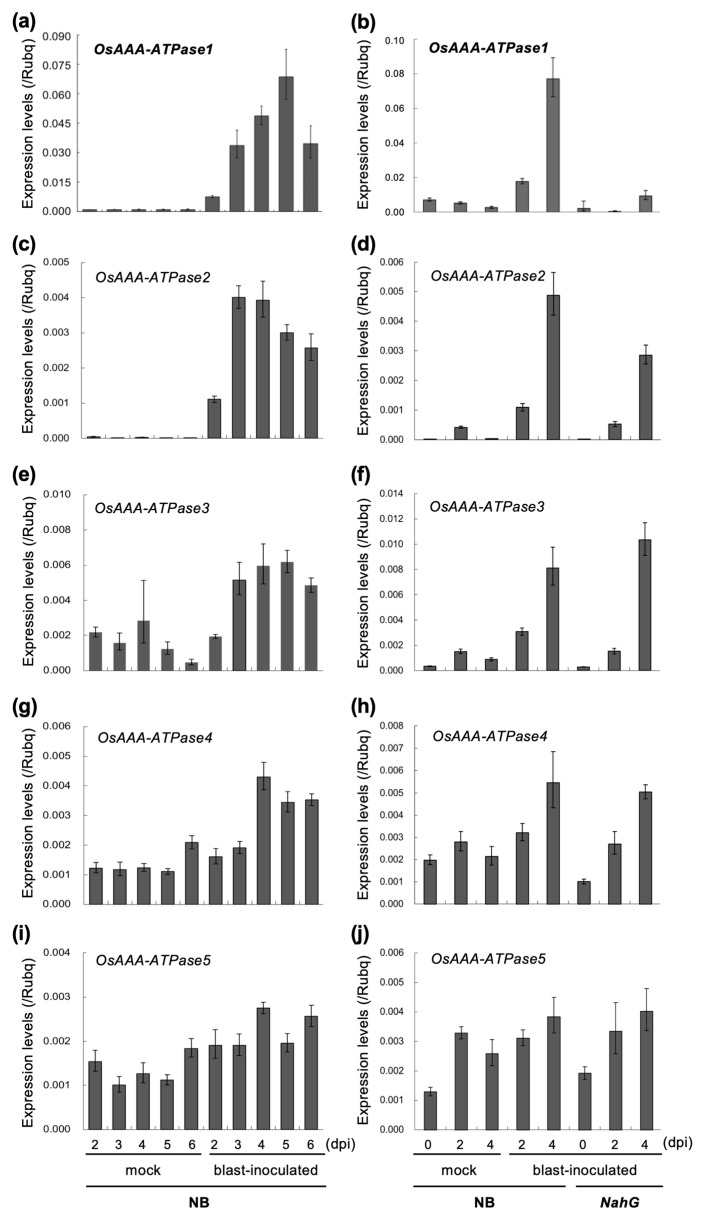
Expression analysis of *OsAAA-ATPase1–5* (**a**–**j**) in response to blast inoculation in Nipponbare (NB) and *narG* rice seedlings. Rice seedlings at the four-leaf stage (three true leaves) were subjected to mock treatment (mock) or blast inoculation (blast-inoculated), and the fourth leaf blades were sampled at indicated days post inoculation (dpi). Data are represented as means ± SDs.

In our previous study, a group of six AAA-ATPase family genes (hereafter *OsAAA-ATPase1–6*; (Table 1; Figure 1) was found to be significantly upregulated in *OsSSI2*-kd rice plants [12], implicating these genes in rice defense activation. From among them, we chose *OsAAA-ATPase1* for more detailed functional characterization in this study, because it showed SA-induced (Figure 2) and SA-dependent blast-induced (Figure 3) transcription responses.

### 2.1. OsAAA-ATPase1 Encodes an AAA-ATPase Family Protein

*OsAAA-ATPase1* was predicted to encode a protein of 520 amino acids with a predicted mass of 58.1 kDa. OsAAA-ATPase1 shared 50%, 37.8%, and 22.3% amino acid sequence identity with previously reported defense-related AAA-ATPase proteins, namely, tobacco NtAAA1 [18], *Arabidopsis* AtOM66 [20], and rice LMR/LRD6-6 [21,22], respectively. Structural analysis revealed that OsAAA-ATPase1 contains consensus motifs that are typical of the AAA-ATPase family; these include the Walker A, Walker B, and SRH motifs (pfam00004; E-value = 4.95 × 10^−17^) (Figure 1a).

To assess how *OsAAA-ATPase1*–*6* are related within the AAA-ATPase gene family, we performed a phylogenic comparison of the proteins predicted to be encoded by *OsAAA-ATPase1–6* and several known rice AAA-ATPase proteins, including LMR/LRD6-6 [21,22], OsCDC48 [27], RuvBL1a [28], RLS3 [29], OsSKD1 [30], OsFtsH5 [31], and RFC5 [32], together with NtAAA1 [18,19] and AtOM66 [20]. OsAAA-ATPase1 was grouped within a subclade of proteins related to plant defense, including OsAAA-ATPase2–6, NtAAA1, and AtOM66, but, unexpectedly, distally with LMR/LRD6-6 (Figure 1b).

### 2.2. OsAAA-ATPase1 is Induced by SA Treatment

Plant hormones have been demonstrated to play important roles in interactions between plants and pathogens. Hence, we examined the transcriptional responses of *OsAAA-ATPase1–6* to the plant hormones abscisic acid (ABA), ACC (an ethylene precursor), BTH (a functional analogue of SA), kinetin (CK, a synthetic cytokinin), auxin (IAA), jasmonic acid (JA), and gibberellic acid (GA). 

*OsAAA-ATPase1* (Figure 2a) and *OsAAA-ATPase3* (Figure 2c) were induced specifically by BTH treatment, and *OsAAA-ATPase2* (Figure 2b) was induced by JA treatment. Meanwhile, *OsAAA-ATPase4* (Figure 2d) and *OsAAA-ATPase5* (Figure 2e) were not specifically induced by any of the hormones, and *OsAAA-ATPase6* (*Os07g0517600*) had no detectable transcription. 

### 2.3. OsAAA-ATPase1 is Induced in Response to Blast Infection in An SA-Dependent Manner

Rice seedlings of non-transformant Nipponbare rice (NB) and of NB expressing the *nahG* gene (*nahG*-rice), at the four-leaf stage, were subjected to blast inoculation. At 2–6 days post inoculation (dpi) of the blast, the fourth leaves were sampled to examine the expression of *OsAAA-ATPase1–5*. 

All of the tested *OsAAA-ATPase* genes clearly showed transcriptional induction in response to blast inoculation (Figure 3c,e,g,i); in particular, *OsAAA-ATPase1* (Figure 3a) and *OsAAA-ATPase2* (Figure 3c) showed a high-fold transcriptional increase from the very low basal levels in the mock treatment. The induction of the genes became evident from 2 dpi and peaked at ca. 3–5 dpi. 

In *nahG*-rice plants, in contrast, the induction of *OsAAA-ATPase1* was mostly attenuated relative to its induction in NB plants in response to blast inoculation (Figure 3b), demonstrating that the induction of this gene depends on the SA-signaling pathway. No appreciable attenuation of gene induction was observed for the other genes (Figure 3d,f,h,j).

### 2.4. OsAAA-ATPase1 is Positively Involved in Blast Resistance

**Figure 4:**
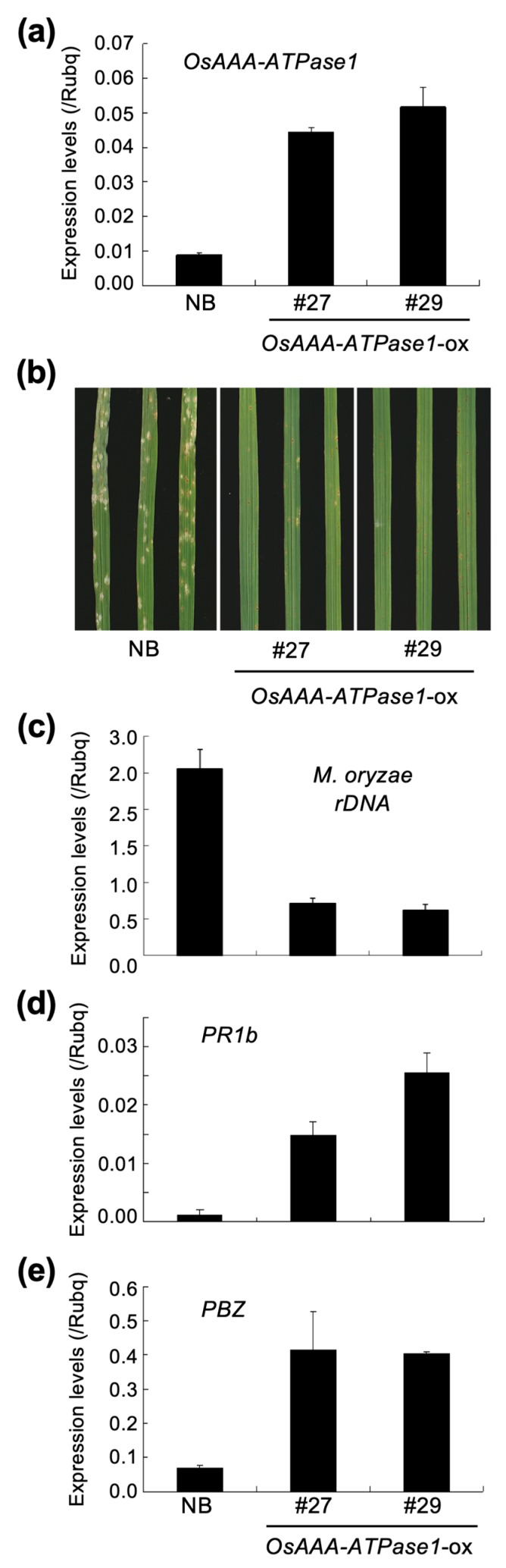
Blast resistance of *OsAAA-ATPase1*-ox plants. (**a**) Expression of *OsAAA-ATPase1*, (**b**) blast lesions on leaf blades, and (**c**) relative fungal growth (*Magnaporthe oryzae rDNA*). (**d**,**e**) Expression of *PR1b* (**d**) and *PBZ1* (**e**), in Nipponbare (NB) and *OsAAA-ATPase1*-ox lines (#27 and #29), respectively, at 7 days post inoculation (dpi). Data are represented as means ± SDs in (**a**,**c**–**e**).

**Figure 5:**
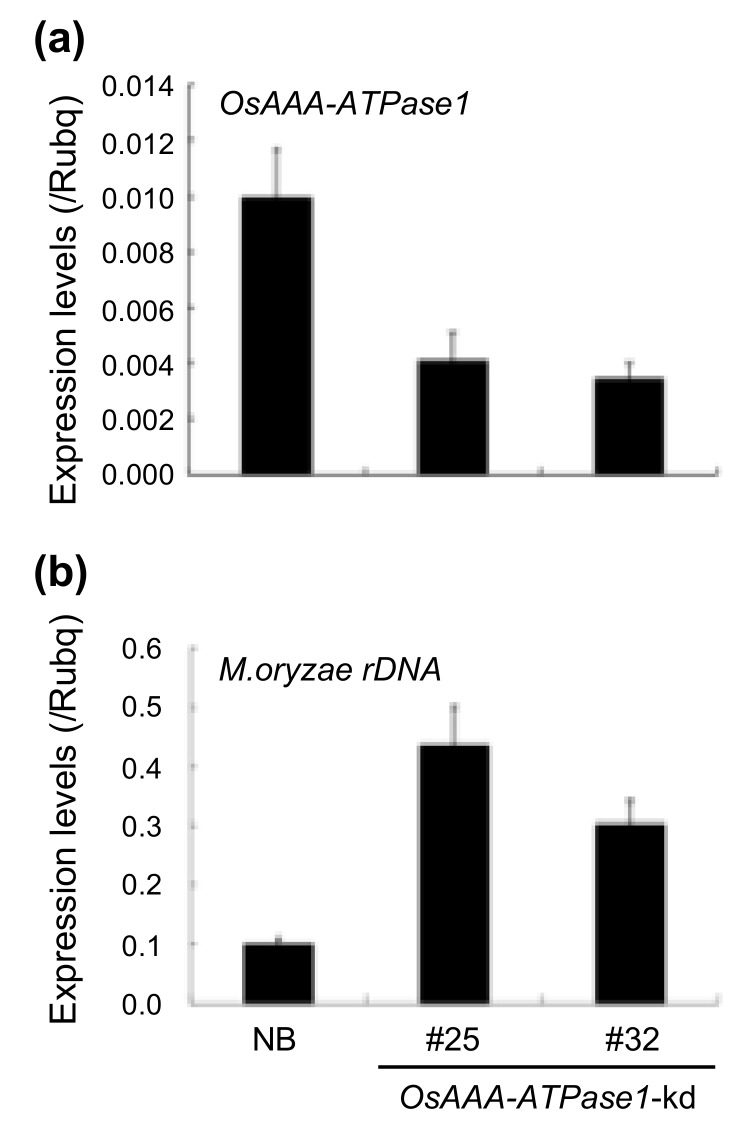
Compromised blast resistance in *OsAAA-ATPase1*-kd plants (#25 and #32). (**a**) Expression of *OsAAA-ATPase1*. (**b**) Relative fungal growth (*M. oryzae rDNA*) in Nipponbare (NB) and *OsAAA-ATPase1*-kd lines (#25 and #32) respectively. Data are represented as means ± SDs.

To gain some insight into the role of *OsAAA-ATPase1* in disease resistance, we generated transgenic rice lines that either overexpressed the gene under maize ubiquitin promoter (*OsAAA-ATPase1*-ox; Figure 4a) or RNAi-suppressed *OsAAA-ATPase1* (*OsAAA-ATPase1*-kd; Figure 5a), and subjected these lines to blast inoculation. In order to reveal the potentially compromised resistance in *OsAAA-ATPase1*-kd plants, a half density of conidia (5 × 10^4^/mL) was used, so as to cause blast disease moderately in NB, but more severely in OsAAA-ATPase1 plants.

Compared with the non-transgenic control plants (NB), *OsAAA-ATPase1*-ox plants (lines #27 and #29) exhibited significantly higher resistance to blast disease, as evidenced by the fact that few susceptible blast lesions appeared on their leaf blades (Figure 4b), and that they had ca. 4-fold less fungal growth (Figure 4c). The enhanced resistance of the *OsAAA-ATPase1*-ox plants is consistent with the large increases in the expression levels of the PR genes, *OsPR1* and *PBZ1* (Figure 4d,e).

Conversely, blast resistance was significantly compromised in *OsAAA-ATPase1*-kd plants (lines #25 and #32): they had ca. 2-fold more fungal growth than the NB control plants (Figure 5b).

### 2.5. OsAAA-ATPase1 Has ATPase Activity and Is Localized in the Cytosol

**Figure 6:**
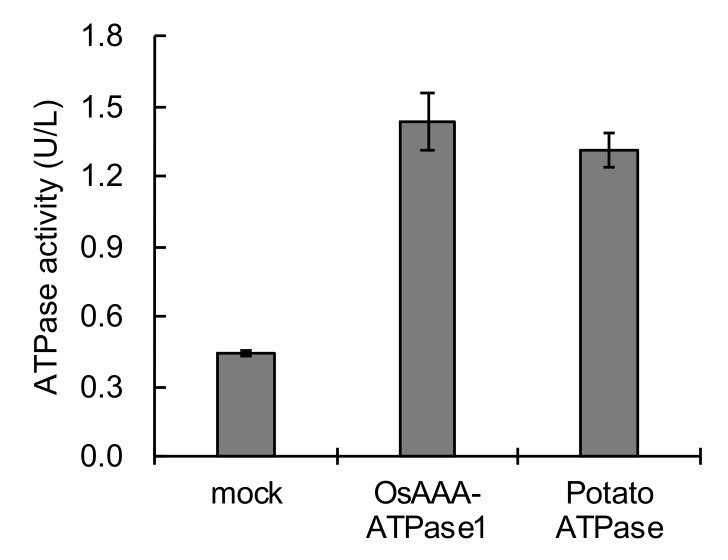
ATPase activity of recombinant OsAAA-ATPase1 protein. Elution buffer of Ni-resin (mock treatment), and an ATPase protein from potatoes, were used negative and positive controls, respectively.

To assess whether OsAAA-ATPase1 protein has ATPase activity, *OsAAA-ATPase1* N-terminal was fused to a His-tag and expressed in *Escherichia coli*, and purified using a high affinity Ni-resin. OsAAA-ATPase1 protein showed an ATPase activity level that was comparable to that of the positive control (potato ATPase) (Figure 6).

**Figure 7:**
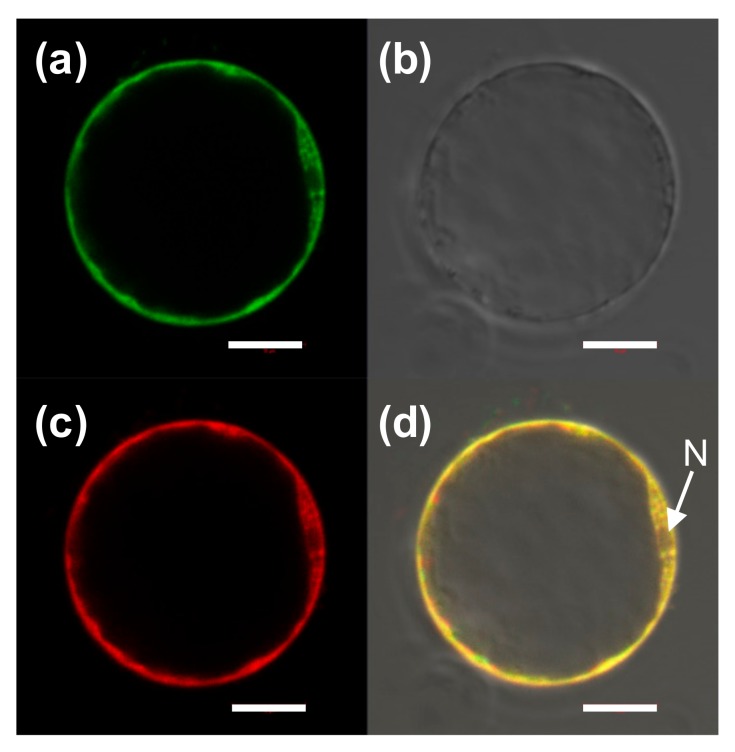
Subcellular localization of OsAAA-ATPase1 in rice protoplasts. (**a**) EGFP-OsAAA-ATPase1, (**b**) blight field image, (**c**) mCherry (cytoplasmic localization), and (**d**) combined image of (**a**–**c**). N, nucleus; bar, 20 μm.

To determine the subcellular localization of OsAAA-ATPase1 in rice cells, the EGFP-OsAAA-ATPase1 fusion protein in the rice protoplast was examined under a confocal microscope. As shown in Figure 7, EGFP-OsAAA-ATPase1 protein was co-localized with a cytosol marker, mCherry signals, indicating that OsAAA-ATPase is predominantly distributed in the cytosol.

## 3. Discussion

AAA-type ATPases constitute a large protein family in a diverse range of organisms, and thus exhibit multiple and diverse cellular functions [15,33]. In plants, AAA-ATPase genes have been implicated in proteolysis [33], male meiosis [34], vacuolar maintenance [35], peroxisome biogenesis [36], morphogenesis [37], leaf senescence [29,38], and stress [28,39] and immune responses [18,19,20,21,22]. In this study, we present a novel rice AAA-ATPase gene member, *OsAAA-ATPase1*. The deduced amino acid sequence of OsAAA-ATPase1 contains consensus motifs that are typical of the AAA-ATPase family; these include the Walker A, Walker B, and SRH motifs (Figure 1a) [15,17,33]. Consistent with this, biochemical analysis confirmed that there was ATPase activity in the recombinant protein of OsAAA-ATPase1 (Figure 6). Phylogenetically, OsAAA-ATPase1 was grouped within a subclade of proteins related to plant defense activation (Figure 1b), which included OsAAA-ATPase2–6 [12], tobacco NtAAA1 [18,19], and *Arabidopsis* AtOM66 [20]. These results suggest that OsAAA-ATPase1 belongs to the AAA-ATPase family. Functional analysis revealed that *OsAAA-ATPase1* is transcriptionally regulated by SA in response to blast infection (Figure 2 and Figure 3). Overexpression or RNAi-mediated suppression of *OsAAA-ATPase1* resulted, respectively, in an increase (Figure 4) or decrease (Figure 5) in blast resistance. Taken together, our results suggest that *OsAAA-ATPase1* plays a positive role in the SA-mediated disease resistance in rice plants.

In relation to plant immune responses, several studies have shown important roles for AAA-ATPase genes. *NtAAA1* was isolated as an HR-induced gene in *Nicotiana tabacum* [18]; was found to be under the control of *N*-gene, ethylene, and jasmonate; and was localized in the cytoplasm. It was also negatively involved in the SA-signaling pathway and pathogen resistance [18,19]. In contrast, *AtOM66* (outer mitochondrial membrane protein of 66 kDa ) is a stress-induced gene; overexpression of this gene increased SA content, accelerated cell death rates, and enhanced resistance to the biotrophic pathogen *Pseudomonas syringae* [20]. Recently, rice *LMR* and *LRD6-6* were map-based cloned from lesion mimic mutants *lmr* and *lrd6-6*, respectively, and were found to be the same gene (*Os06g0130000*). LMR/LRD6-6 was shown to be localized in the multivesicular bodies (MVBs) and was negatively involved in rice immunity and cell death [21,22]. Mutation in this gene (*lmr* and *lrd6-6*) resulted in constitutive expression of *PR1* and *PBZ1*, and enhanced resistance to rice blast and bacterial blight diseases; however, no difference in SA content was determined [21,22]. By comparison, it seems that OsAAA-ATPase1 plays a role distinct from those previously reported, with respect to its association with SA-regulation and HR, its subcellular localization, and its promotion of disease resistance. Thus, our findings provide novel insights into SA-regulated defense activation in rice. Meanwhile, OsAAA-ATPase1 showed a close phylogenetic association with AtOM66 (Figure 1b); both proteins play a positive role in the SA-signaling pathway, suggesting that they may share a common cellular function.

Plants produce a variety of FAs and their derivatives, some of which have been shown to play important roles in defense activation [40,41]. In the *Arabidopsis ssi2* mutant, disruption of *SSI2*, which encodes an FA desaturase, results in an increase in the 18:0 FA content, which in turn remarkably increases SA content, PR gene expression, and resistance against multiple pathogens [42]. Similar defense-related phenotypes were observed following suppression of *SSI2*-orthologs in soybean (*GmSACPD-A/-B*) [11], rice (*OsSSI2*) [12], and wheat (*TaSSI2*) [13,14]. These results strongly suggest that *SSI2* and its orthologs serve as valuable susceptibility gene (*S* gene) resources for the development of crop cultivars with resistance to multiple pathogens, by employing targeted mutation and genome editing technologies [43,44,45]. In order to make such successful use of these genes in resistance breeding, it is important to understand the molecular mechanisms underlying the defense activation. In *Arabidopsis*, a mutation in the GTPase nitric oxide associated 1 (*NOA1*) gene partially restored the *ssi2* phenotype, whereas double mutations in NOA1 and either one of the two nitrate reductase isoforms (NIA1 and NIA2) completely restored the *ssi2* phenotypes; this indicates that nitric oxide (NO) is required for constitutive defense in the *ssi2* mutant [46,47]. Nevertheless, little has been reported regarding the molecular basis of defense activation in *OsSSI2*-kd rice plants. We previously identified a group of six AAA-ATPase genes (*OsAAA-ATPase1–6*) that were upregulated in *OsSSI2*-kd rice plants [12]. In this study, all of these genes tested were induced in response to blast inoculation (Figure 3), suggesting that they each play a role in resistance to blast fungus. In contrast, *OsAAA-ATPase1–5* each exhibited a distinct induction pattern in response to different plant hormone treatments (Figure 2); *OsAAA-ATPase1* and *OsAAA-ATPase3* were induced by SA, *OsAAA-ATPase2* mainly by JA, and *OsAAA-ATPase4* and *OsAAA-ATPase5* slightly by the CK treatment. These results suggest that there is functional differentiation among the *OsAAA-ATPase1–6* genes downstream of *OsSSI2* in disease resistance. Moreover, although both *OsAAA-ATPase1* and *OsAAA-ATPase3* were induced by SA treatment, only the induction of *OsAAA-ATPase1* was attenuated following blast infection in *nahG*-rice plants (Figure 3). One possible explanation for this is that *OsAAA-ATPase3* may be more sensitive to SA, allowing it to be induced even by a residual increase in the SA-signaling level in *nahG*-rice plants.

## 4. Materials and Methods 

### 4.1. Plant Materials and Growth Conditions

Japonica type rice cultivar Nipponbare (*Oryza sativa* L.) was grown in commercial nursery soil (Bonsol Number 2; Sumitomo Chemical Corp., Tokyo, Japan) in a greenhouse at 28 °C (day)/23 ° C (night) with ca. 50% relative humidity.

### 4.2. Plasmid DNA Construction and Rice Transformation

The cDNA clone for *OsAAA-ATPase1* was provided by the Rice Genome Resource Center, Japan (accession number: AK070731). To construct a plasmid for constitutive expression of *OsAAA-ATPase1* under the maize ubiquitin promoter, a DNA fragment containing a 91 bp upstream sequence followed by the full coding sequence of *OsAAA-ATPase1* (nucleotides 2–1655) was amplified by PCR and cloned into the pUCAP/Ubi-NT vector, as previously described [5]. To construct a plasmid for *OsAAA-ATPase1* RNAi (*OsAAA-ATPase1*-kd), part of the 3′-UTR (nucleotides 1543–1845) of *OsAAA-ATPase1* cDNA was amplified by PCR and cloned into the pANDA vector, as previously described [48,49].

Nipponbare rice plants were transformed by an *Agrobacterium tumefaciens* (strain EHA105) mediated technique, as described earlier [50]. Transgenic rice lines expressing *nahG* from *Pseudomonas putida* under the control of a double 35S promoter (*nahG*-rice) were generated using the plant expression construct previously described in Yang et al. [51].

### 4.3. Chemical Treatments

All stock solutions were prepared at a concentration of 100 mmol/L. Indole-3-acetic acid (IAA; Sigma, St. Louis, MO, USA), gibberellin A3 (GA_3_; Wako, Osaka, Japan), abscisic acid ((±)-*cis*-*trans*, ABA; Sigma), and methyl jasmonate (ME-JA; Wako, Saitama, Japan) were dissolved in ethanol. Kinetin (Sigma) and benzothiadiazole *S*-methyl ester (BTH; Wako) were dissolved in dimethyl sulfoxide (DMSO); and 1-aminocyclopropane-1-carboxylic acid (ACC; Sigma) and sodium salicylate (SA; Nacalai Tesque, Tokyo, Japan) were dissolved in H_2_O. The solvents did not exceed a final concentration of 0.1% in the solutions used for plant treatments, and had no effect on the expression of the rice genes examined in this study.

For plant treatments, rice seedlings at the four-leaf stage (three true leaves) were transferred to a container containing each of the plant hormone solutions at 50 μM. The rice seedlings were further grown for 1 day, and fourth leaf blades were stored in liquid nitrogen for RNA preparation.

### 4.4. Protein Expression, Purification, and ATPase Assay

The *OsAAA-ATPase1* sequence was amplified by PCR and cloned into the sites between BglI and HidIIIp of pET32a (Novagen) as a His-tag fusion protein; then, that was transfected into the *Escherichia coli* Origami strain BL21(Lys). The set of primers used was as follows: OsAAA1BglII 5′-GTAGATCTCTTGAGACAAATGGAGGCGACG-3′; OsAAA1HindIII 5′-GCTAAGCTTCTACTTATCCTTCCCGACCAC-3′. Expression of the protein was induced for 4 h at 25 °C with 0.5 mmol/L isopropyl β-d-1-thiogalactopyranoside. *Escherichia coli* cells were pelleted by centrifugation, resuspended in lysis buffer (20 mmol/L Tris-HCl pH 7.4, 0.1 M NaCl, 10 mmol/L imidazole), and sonicated. After the cell debris was removed by centrifugation (12,000 × g, 10 min, 4 °C), the supernatant was loaded onto a High Affinity Ni-Charged Resin (GE Healthcare, Buckinghamshire, UK), washed with washing buffer (20 mmol/L Tris-HCl pH 7.4, 0.1 M NaCl, 10 mmol/L imidazole), and eluted with elution buffer (20 mmol/L Tris-HCl pH 7.4, 0.1 M NaCl, 180 mmol/L imidazole). ATPase activity was measured by the malachite green-based colorimetric method using the QuantiChrom^TM^ ATPase/GTPase activity assay kit (Sigma-Aldrich, St. Louis, MO, USA). The elution buffer was used as the negative control, and an ATPase from potatoes (Sigma-Aldrich, St. Louis, MO, USA) was used as the positive control. One unit is defined as the amount of enzyme that catalyzes the production of 1 μM of free phosphate per minute under the assay conditions.

### 4.5. Subcellular Localization

For subcellular localization of OsAAA-ATPase1, the plasmid pSAT6-AFP-C1-OsAAA1 was transformed into protoplasts prepared from etiolated seedlings as previously described [52]. As a control for cytoplasmic localization, the pSAT-mCherry construct was co-transformed. Fluorescence was examined under a confocal microscope (Leica Microsystems, Wetzlar, Germany) 16 h after transformation.

### 4.6. Pathogen Culture and Inoculations

Culture and inoculation of the blast fungus *M. oryzae* (compatible race 007.0) was conducted essentially as previously described [12], with slight modifications. Briefly, the fungus was grown on an oatmeal agar medium (30 g/L oatmeal, 5 g/L sucrose, and 16 g/L agar) at 26 °C for 10–12 day. After removing the aerial hyphae by washing with distilled water and a brush, conidia formation was induced by irradiation under continuous black blue light (FL15BLB; Toshiba, Osaka, Japan) at 24 °C for 3 day. The conidia were suspended in 0.02% Silwet L-77 (a non-ionic surfactant; Nihon Unica, Tokyo, Japan) at a density of 10^5^/mL, and were sprayed onto rice plants at the four-leaf stage. After incubation in a dew chamber at 24 °C for 24 h, the rice plants were moved back to the greenhouse. 

Disease development was evaluated by determining the *M. oryzae* genomic 28S *rDNA* [26] by qRT-PCR [5,6], 6–7 dpi. At least 20 plants were used for each disease assay.

### 4.7. RNA Analyses

Total RNA was isolated from leaf blades of the 4th leaves of rice seedlings using the Trizol reagent (Invitrogen, Carlsbad, CA, USA). Quantitative RT-PCR (qRT-PCR) was performed on a Thermal Cycler Dice TP800 system (Takara Bio, Tokyo, Japan) using SYBR premix Ex Taq mixture (Takara Bio) as previously described [5]. The primer sequences used for qRT-PCR are listed in Table 1.

### 4.8. Amino Acid Sequence Alignment and Phylogenetic Analysis

The protein sequences were retrieved from the rice annotation project database (rap-db) and aligned using Clustal-X software, and the tree was constructed using iTOL software [53].

## 5. Conclusions

In this study, we present a novel AAA-ATPase member gene, *OsAAA-ATPase1*, one of the six AAA-ATPase genes upregulated in *OsSSI2*-kd rice plants [12]. Functional analysis revealed that *OsAAA-ATPase1* is transcriptionally regulated by SA, and plays a positive role in the SA-mediated disease resistance in rice plants. Our findings provide novel insights into SA-regulated defense activation in rice, and the molecular basis of defense activation in *OsSSI2*-kd rice plants.

## Figures and Tables

**Table 1:** Genes and primer sequences used for qRT-PCR analysis.

Gene Name (RAP+DB ID)	Primer Sequences (5’→3’)	References
***OsAAA-ATPase1*** Os03g0802500	AGTGGTTGCTAGCTTCTCGT ACAACATGTGGTCAAATTATTCCA	[12]
*OsAAA-ATPase2* Os01g0297200	CTAGGTTCTGCGATGGACAC CTCCTTTGCAATTGTTCCAC	[12]
*OsAAA-ATPase3* Os06g0697600	GTTGTGATCGTGTCATGGTTGCG CAGAAAGCCACACACCATTGC	[12]
*OsAAA-ATPase4* Os02g0697600	TTGCCTGAACGGCCAGGTGAT CCCATGTAAGGGTAAGGATTGC	[12]
*OsAAA-ATPase5* Os02g0706500	GTTCCATCTCTTTGCCTGTAGC CATGCGCATCTCAGTCTTACC	[12]
*OsAAA-ATPase6* Os07g0517600	TCAGTGGCCTCGTCGAGTTC CTACTTGCCTGCTTCACACAT	[12]
*OsPR1b* Os01g0382000	ACGGGCGTACGTACTGGCTA CTCGGTATGGACCGTGAAG	[23]
*PBZ1* Os12g0555000	GCGTTTGAGTCCGTGAGAGT TCACCCATTGATGAAGCAAA	[24]
Rubq1 Os06g0681400	GGAGCTGCTGCTGTTCTAGG TTCAGACACCATCAAACCAGA	[25]
*M. oryzae* 28S rDNA	ACGAGAGGAACCGCTCATTCAGATAATT TCAGCAGATCGTAACGATAAAGCTACTC	[26]
